# Supplementary material for: Ultrasensitive rapid cytokine sensors based on asymmetric geometry two-dimensional MoS2 diodes
Source: Nat Commun. 2022 Dec 19;13:7593. doi: 10.1038/s41467-022-35278-2 (PMC9763493; doi:10.1038/s41467-022-35278-2)
Supplement: Supplementary file 1 — Supplementary Information [file 41467_2022_35278_MOESM1_ESM.pdf]

Ultrasensitive rapid cytokine sensors based on asymmetric geometry two-dimensional MoS_2 diodes

Thushani De Silva[1], Mirette Fawzy[2], Amirhossein Hasani[1], Hamidreza Ghanbari[1], Amin Abnavi[1], Abdelrahman Askar[1], Yue Ling[1], Mohammad Reza Mohammadzadeh[1], Fahmid Kabir[1], Ribwar Ahmadi[1], Miriam Rosin[3], Karen L. Kavanagh[2], Michael M. Adachi[1]

[1]School of Engineering Science, Simon Fraser University, Burnaby V5A 1S6, BC, Canada

[2]Department of Physics, Simon Fraser University, Burnaby V5A 1S6, BC, Canada

[3]Department of Biomedical Physiology and Kinesiology, Simon Fraser University, Burnaby V5A 1S6, BC, Canada

Corresponding author: mmadachi@sfu.ca

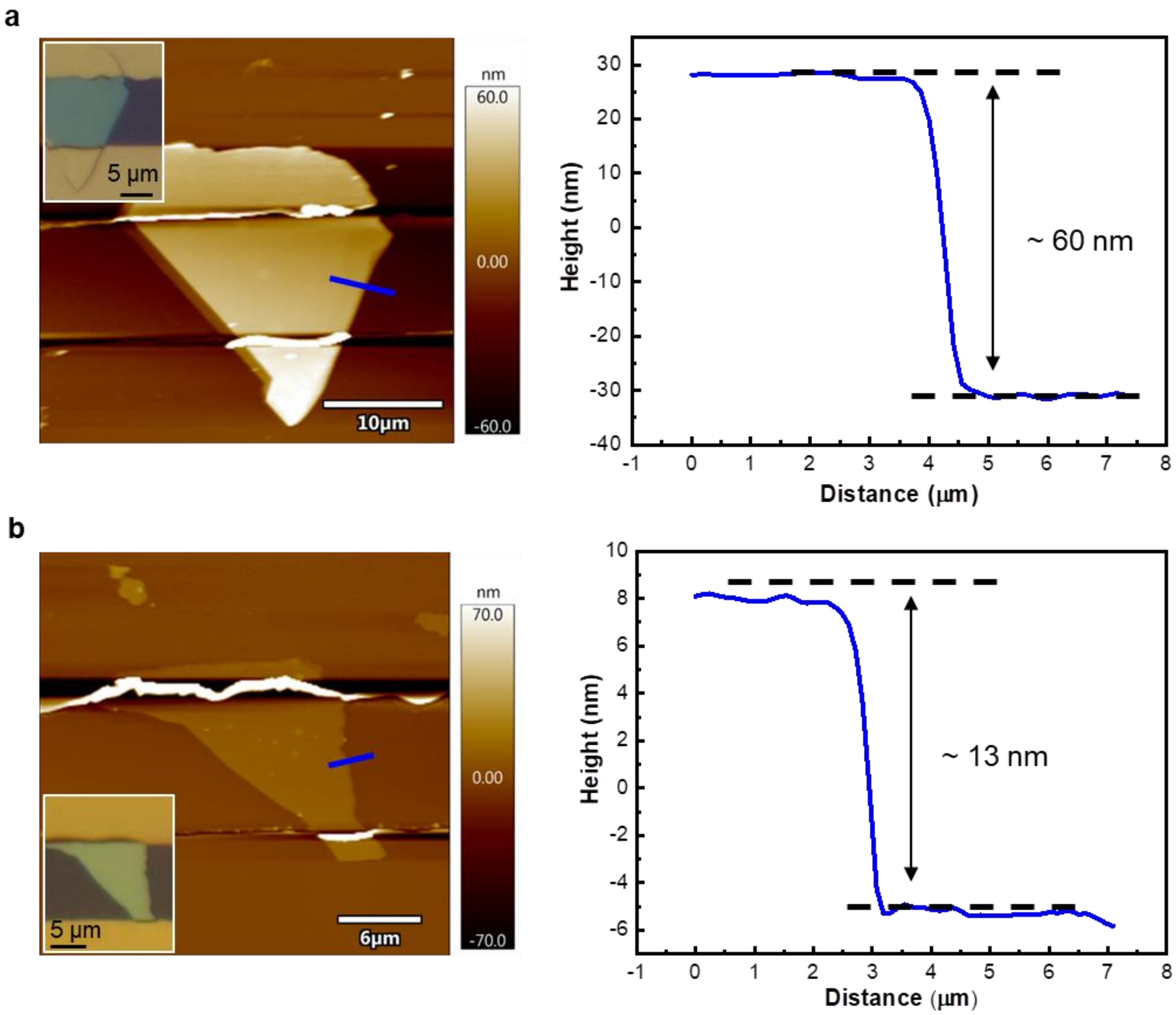

Supplementary Figure 1: AFM height images (left) with blue lines indicating profiles (right) for two typical flakes of colors a blue and **b** green. Optical images are found in the insets (left).

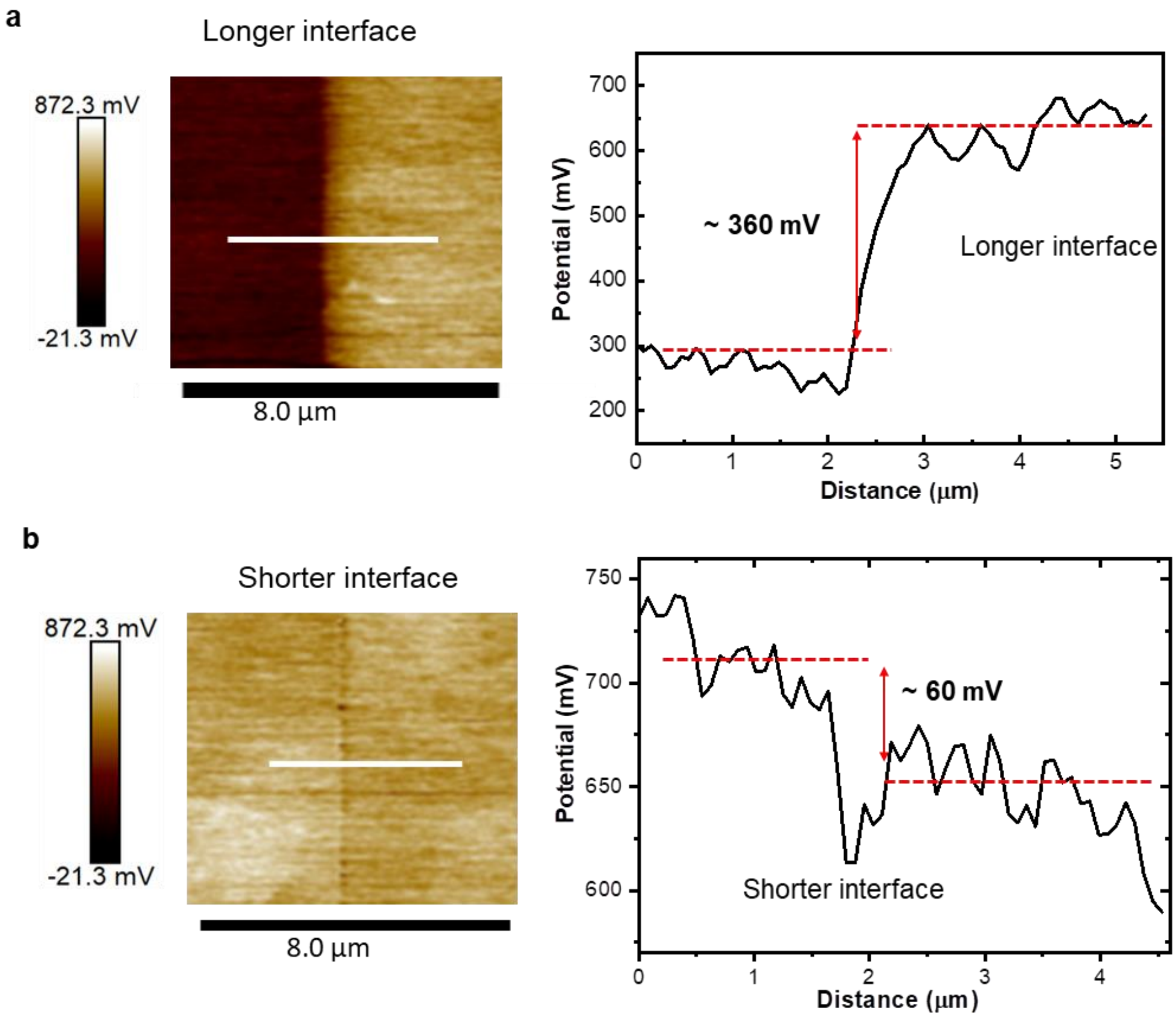

Supplementary Figure 2: Surface potential maps across contact interfaces, images (left) with profile along white lines (right) for a longer **b** shorter interface.

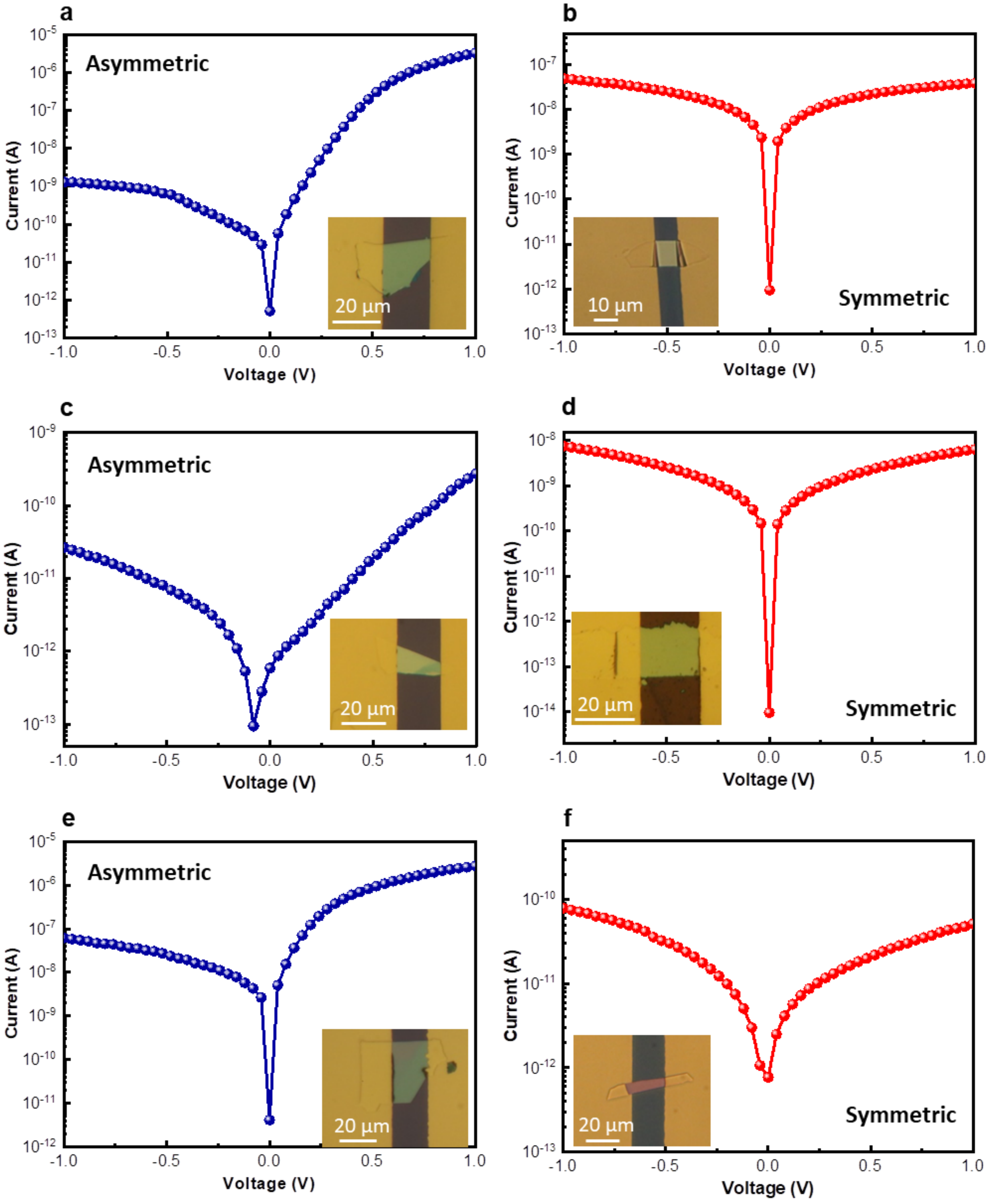

Supplementary Figure 3: Current-voltage curves from, **a, c, e**, three asymmetric devices with a noticeable rectification behavior. **b, d, f,** three symmetric devices with a very low or negligible rectification behavior.

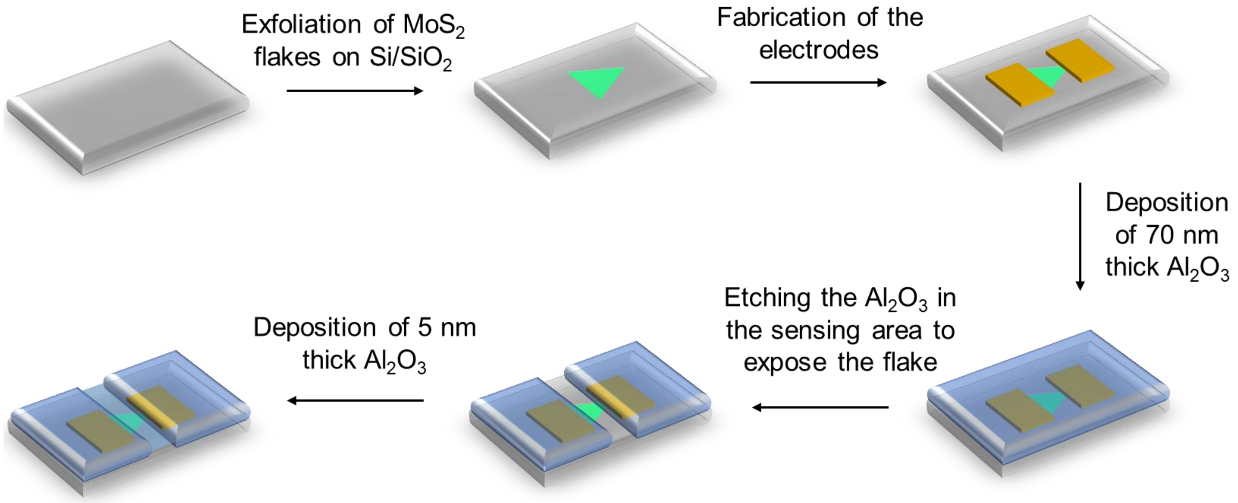

Supplementary Figure 4: Fabrication process of the cytokine sensor. The MoS_2 flakes are first exfoliated onto a clean Si/SiO_2 substrate. Then, 10 nm Cr and 50 nm Au were deposited using thermal evaporation for electrical contacts, on a selected flake following photolithography. Next, a 70 nm thick Al_2O_3 layer was deposited for surface passivation. In order to expose the flake in the sensing area, a narrow rectangular area over the flake was cleared of Al_2O_3. Then, a thinner Al_2O_3 with a thickness of 5 nm was deposited on the substrate, facilitating the functionalization of the aptamers on the sensing area.

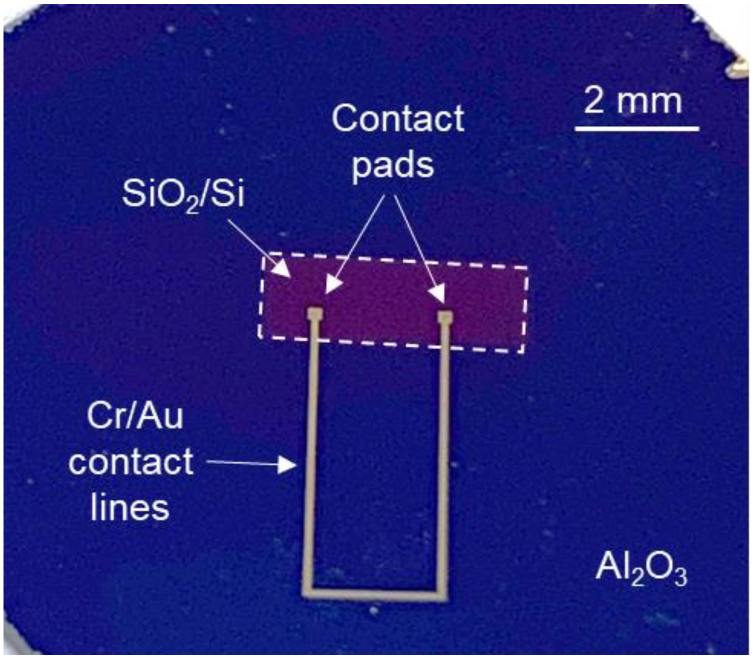

Supplementary Figure 5: An Optical Image of the sensor. The sensor has longer contact lines with the contact pads far away from the sensing area. A layer of Al_2O_3 is deposited everywhere (blue area on the substrate) except inside the dotted rectangle. The Al_2O_3 inside the dotted rectangle (purple area) has been etched completely to facilitate a good electrical connection between the contact pads and the probes station.

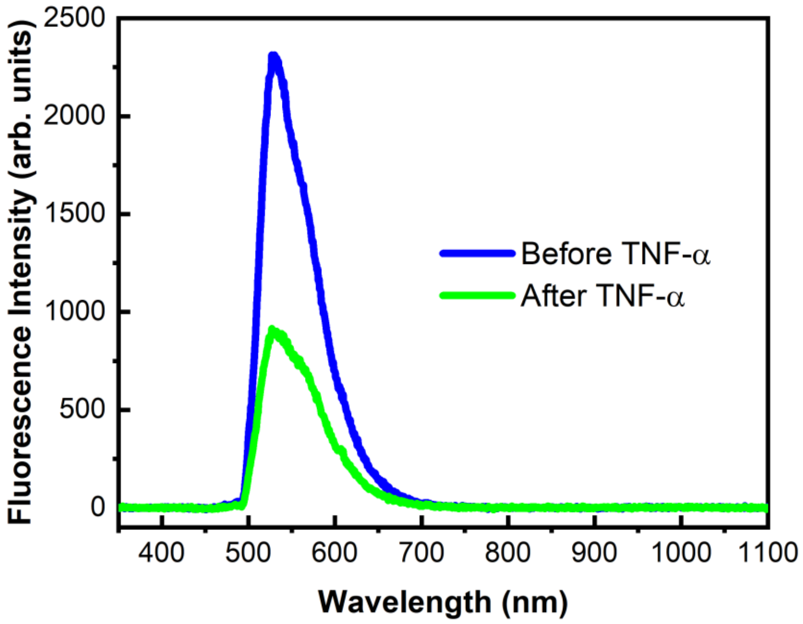

Supplementary Figure 6: Fluorescence spectrum during cytokine interaction. The fluorescence spectrum of the aptamer functionalized surface before and after the interaction with TNF-α cytokines at a concentration of 58.5 nM.

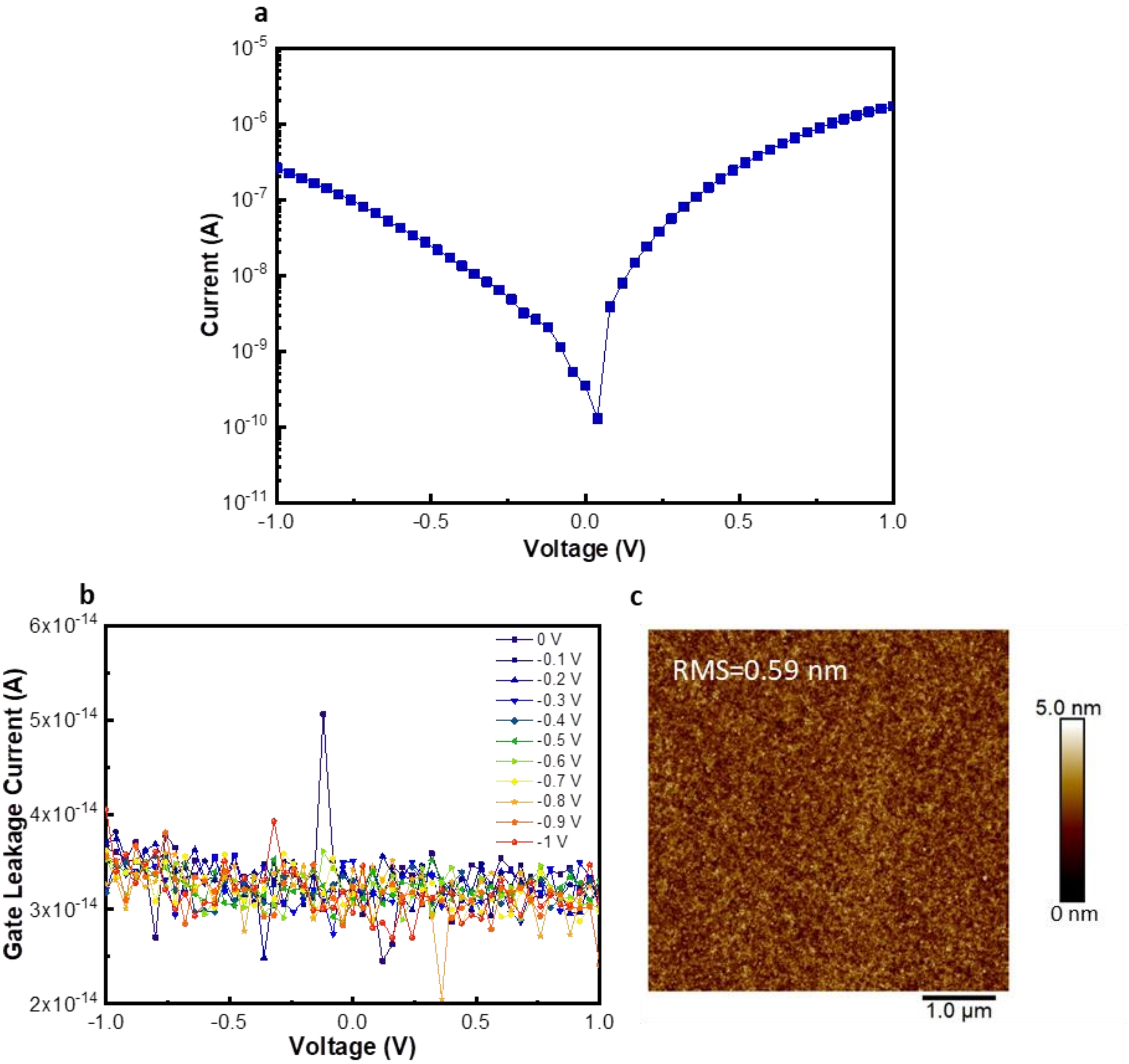

Supplementary Figure 7: I-V and AFM measurements on stability of the biosensor. a I-V response of the cytokine diode sensor (shown in Fig. 4c) in air. **b** Gate leakage current response for the drain-source current (I_{DS}) versus drain-source voltage (V_{DS}) for different applied gate voltages (V_{GS}) ranging from 0 V to -1 V shown in Fig. 4c. **c** AFM height image on the 5 nm Al_2O_3 film over the sensing area (in the trench). AFM scans were performed four times on the same device (which gave similar results) and one height scan is presented.

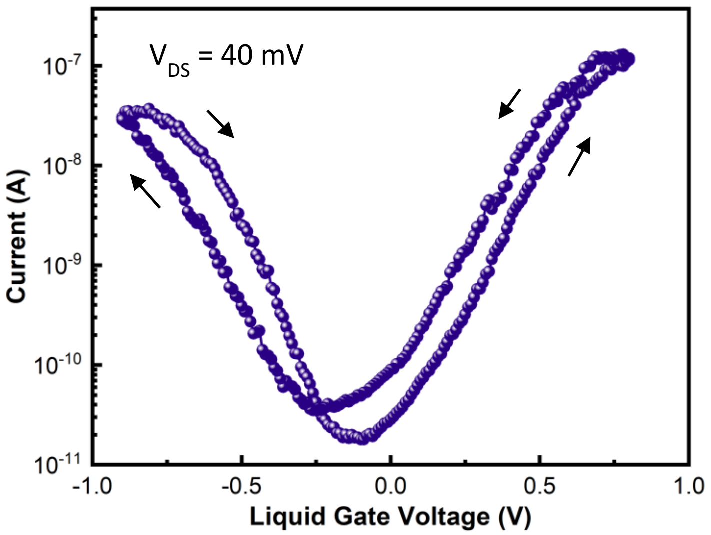

Supplementary Figure 8: Transfer curve of the functionalized biosensor. The dual sweep showing the ambipolar behavior. The hysteresis is related to the scanning speed of the gate bias which was 10 mV/sec.

During initial trials, the cytokine sensor was fabricated with a one-step Al_2O_3 deposition where several devices were fabricated with different Al_2O_3 thicknesses varying from 15 nm to 40 nm (on the sensing area). However, during the measurement for cytokine detection, it was noticed that some MoS_2 flakes were being oxidized. We assume that this is due to the failure of the Al_2O_3 passivation layer. As a solution, a two-step Al_2O_3 passivation was employed where a thicker layer (75 nm) was deposited to cover the electrode area and a thinner layer (5 nm) was deposited over the sensing area (as shown in the schematic of the fabrication process).

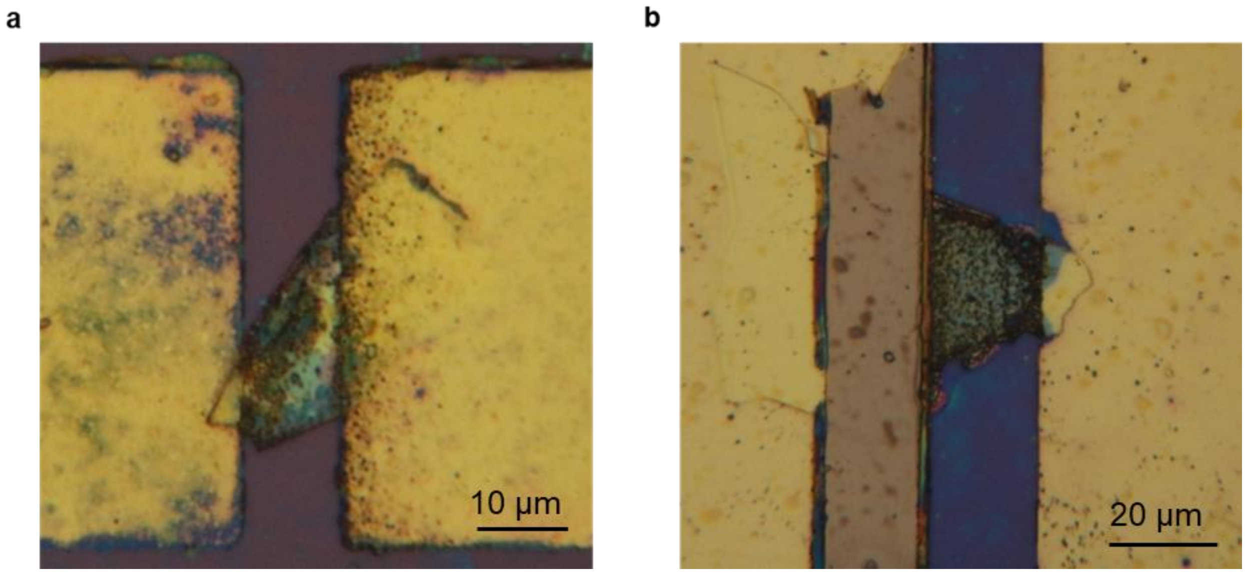

Supplementary Figure 9: Optical images of degraded flakes during measurements in PBS. A clear discoloring can be seen on these two examples of MoS_2 flakes and electrode area near the flake likely due to oxidization.

Each experiment was repeated a few times on different devices. However, due to the nature of the biological samples, we could only repeat one set of experiments, one time for each sensor[1]. Furthermore, a major disadvantage of using exfoliated flakes is that we do not have control over the dimension and the shape of the flakes. Therefore, each device possessed a different initial rectification factor (*RF*) and current levels, resulting in a varying response from device to device. Due to this reason, the standard deviation calculations are not valid since the responses were not at the same scale. Nevertheless, we were able to confirm the trend of the cytokine sensor by repeating the test on several devices were with the increase of the cytokine concentration, an increase in the figure of merit (Normalized *RF*) was observed. However, with improved synthesis techniques to achieve uniform devices with similar performance, we believe that one calibration in PBS would be sufficient for all the devices.

Even though the two-step Al_2O_3 deposition method has proven to provide a better passivation since it allows to have a thicker layer (75 nm) over the electrode area, some of the devices fabricated incorporating the one-step method still survived during the cytokine measurements and showed the same trend overall. The figures below illustrate the repeatability of the cytokine diode senor observed for the devices fabricated using two-step Al_2O_3 deposition method and the initial one-step Al_2O_3 deposition method (with different Al_2O_3 layer thicknesses over the sensing area).

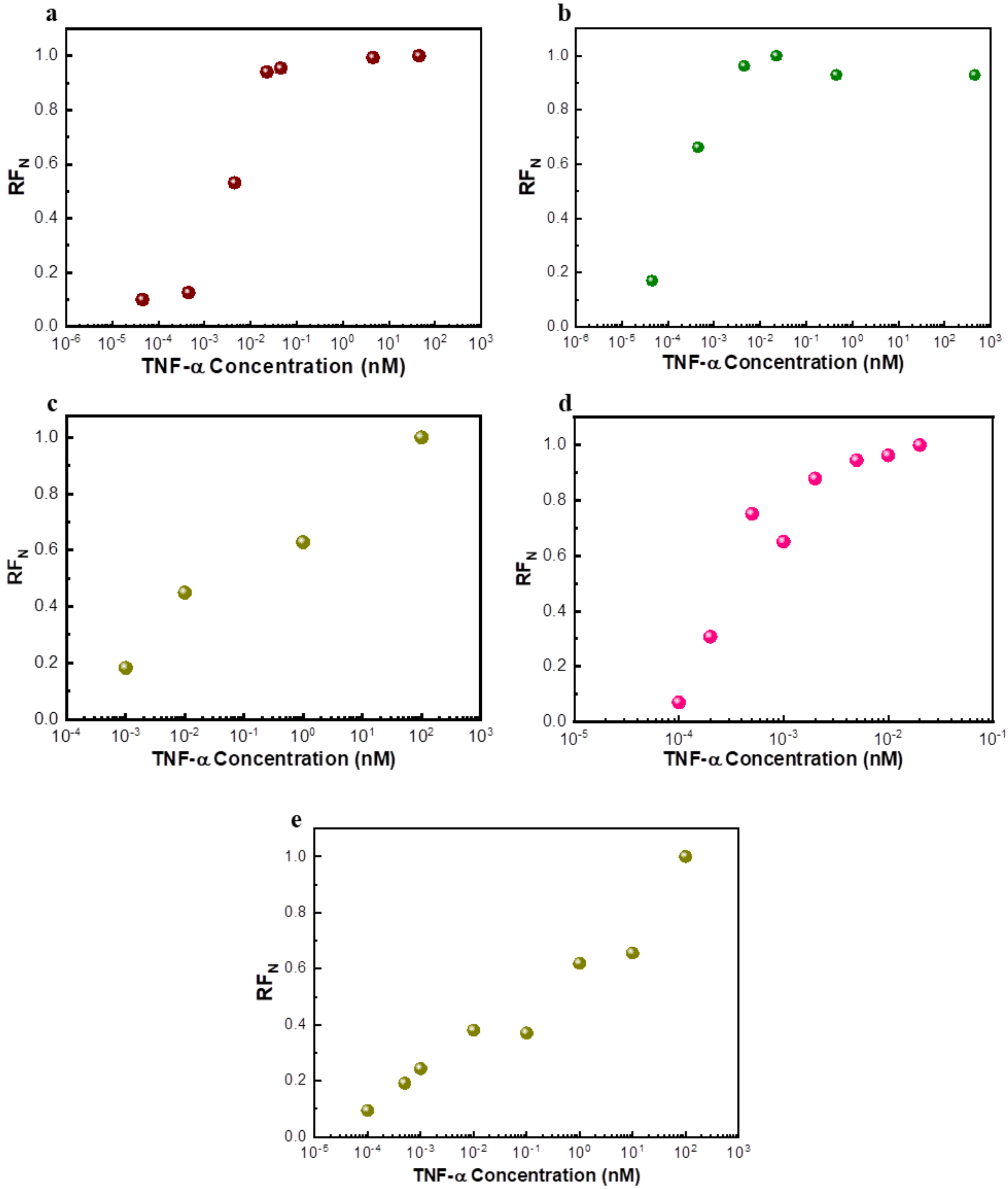

Supplementary Figure 10: Variation of the normalized RF with increase of the TNF-α concentration. The data has been normalized so that the response in pure PBS was set to zero and the highest response is set to 1. Response for a diode sensor fabricated with **a** the two-step Al_2O_3 deposition method where the Al_2O_3 thickness over the sensing area is 5 nm. **b** same method as a. **c** 15 nm thick one-step Al_2O_3 deposition method. This device was only tested for

the 4 cytokine concentrations (starting at 0.001 nM) as shown in the graph but still shows the same trend. **d** 20 nm thick one-step Al_2O_3 deposition method. This device was mainly tested for lower concentrations starting from 0.0001 nM to 0.01nM which shows the same trend. **e** 40 nm thick one-step Al_2O_3 deposition method, showing the general trend.

Supplementary References

1 Fathi-Hafshejani, P. *et al.* Two-dimensional-material-based field-effect transistor biosensor for detecting COVID-19 Virus (SARS-CoV-2). *ACS nano* **15**, 11461-11469 (2021).
